# Magnetic Resonance Imaging–Transrectal Ultrasound Fusion‐Targeted Biopsy Improves the Diagnostic Efficacy of Overall and Clinically Significant Prostate Cancer

**DOI:** 10.1111/iju.70334

**Published:** 2025-12-26

**Authors:** Yusuke Fukiage, Minekatsu Taga, Mahiro Inamura, Kimika Kaeriyama, Nobuki Tanaka, Wonseok Seo, Tadashi Kakitsuba, Sahoko Shimada, Nodoka Okubo, Takafumi Kabuto, Manami Tsutsumiuchi, Yoshinaga Okumura, Akifumi Muramoto, Masaya Seki, So Inamura, Naoki Terada

**Affiliations:** ^1^ Department of Urology, Faculty of Medical Sciences University of Fukui Eiheiji Japan; ^2^ Department of Pathology, Faculty of Medical Sciences University of Fukui Eiheiji Japan

**Keywords:** image‐guided biopsy, interventional ultrasonography, magnetic resonance imaging, needle biopsy, prostatic neoplasms

## Abstract

**Objectives:**

As the diagnostic efficacy and safety of magnetic resonance imaging (MRI)–transrectal ultrasound (TRUS) fusion‐targeted transrectal biopsy for prostate cancer (PCa) detection have already been demonstrated when compared with those of conventional systematic biopsy, we aimed to further evaluate them.

**Methods:**

In this retrospective study, we included patients who underwent MRI–TRUS fusion‐targeted transrectal biopsy combined with systematic biopsy between 2022 and 2024. The detection rates for overall and clinically significant PCa and adverse event (≥ grade 3) rates were assessed. The results were compared with those of conventional systematic biopsies using propensity score matching.

**Results:**

In 223 patients who underwent MRI–TRUS fusion biopsy combined with systematic biopsy (initial biopsy, 161; repeat biopsy, 62), the median prostate‐specific antigen level was 8.0 ng/mL; the median prostate volume was 35 mL. The overall, clinically significant, and insignificant PCa detection rates were 67%, 60%, and 8%, respectively. After propensity score matching, detection rates were significantly higher in the MRI–TRUS fusion group than in the control group for overall PCa (66% vs. 49%, *p* = 0.005) and clinically significant PCa (58% vs. 41%, *p* = 0.005), but were not significantly different for clinically insignificant PCa (8% vs. 8%, *p* = 1.000). The adverse event rate (≥ grade 3) was not significantly different between the groups (3% vs. 3%, *p* = 1.000).

**Conclusions:**

Compared with conventional biopsy, MRI–TRUS fusion‐targeted transrectal biopsy combined with systematic biopsy could effectively detect clinically significant PCa without increasing detection of clinically insignificant PCa and adverse event rates.

**Abbreviations**
csPCa: clinically significant prostate cancer
CZ: central zone
EZR: Easy R
IQR: interquartile range
ISUP: International Society of Urological Pathology
mpMRI: multiparametric magnetic resonance imaging
MRI: magnetic resonance imaging
PCa: prostate cancer
PI‐RADS: Prostate Imaging Reporting and Data System
PSA: prostate‐specific antigen
PZ: peripheral zone
TRUS: transrectal ultrasound
TZ: transitional zone

## 1 Introduction

Prostate cancer (PCa) is one of the most frequently diagnosed malignancies in men and represents a major public health concern worldwide; it is the second most common cancer and fifth leading cause of cancer‐related deaths among men globally [1]. Although the incidence of PCa varies geographically, accurate diagnosis is critical for detecting clinically significant PCa (csPCa) while avoiding overtreatment of clinically insignificant PCa (cisPCa).

Conventional systematic biopsies performed transrectally under transrectal ultrasound (TRUS) guidance have long been the standard diagnostic approach. However, conventional systematic biopsy has limited sensitivity for PCa, leading to unnecessary biopsies in patients who do not have the disease. A missed csPCa diagnosis may delay effective treatment and worsen outcomes, whereas obtaining more biopsy cores increases the detection of cisPCa, potentially triggering patient anxiety, overtreatment, and high healthcare costs [2, 3].

In recent years, multiparametric magnetic resonance imaging (mpMRI) has become a critical tool for the diagnosis of PCa [4]. As a detailed anatomical and functional imaging technique, mpMRI enables the precise localization of suspicious lesions. Additionally, the Prostate Imaging Reporting and Data System (PI‐RADS) standardizes the evaluation and reporting of mpMRI findings, thereby enhancing the reliability and reproducibility of PCa detection [5]. MRI–TRUS fusion‐targeted biopsy combines mpMRI results with real‐time TRUS imaging to enable the precise sampling of suspected PCa lesions. This approach, whose usefulness compared with conventional systematic biopsy has already been widely recognized, improves csPCa detection rates while reducing cisPCa detection rates. Landmark studies, such as the PRECISION trial, have highlighted the diagnostic advantages of MRI–TRUS fusion‐targeted biopsy, particularly in the initial biopsy of biopsy‐naïve patients [6].

Recent evidence indicates that combining MRI–TRUS fusion‐targeted biopsy improves cancer detection rates, particularly for high‐probability lesions (PI‐RADS ≥ 4). However, its diagnostic performance for PI‐RADS 3 lesions has been reported to be lower [7]. Moreover, the detection rates tend to be lower in repeat‐biopsy cases and for lesions in the transitional/central zones (TZ/CZ) than in the peripheral zone (PZ) [8, 9, 10].

Therefore, in this study, we investigated the diagnostic efficacy and safety of MRI–TRUS fusion‐targeted transrectal biopsy combined with systematic biopsy for the detection of PCa, including csPCa and cisPCa. The detection rates were evaluated for the initial or repeat biopsy, each prostate lesion, and each PI‐RADS category and compared with those obtained from previously performed conventional systematic biopsies. This study approach aims to provide additional insights into the diagnostic utility of MRI–TRUS fusion‐targeted transrectal biopsy in routine clinical practice.

## 2 Patients and Methods

### 2.1 Patients

This study included patients who underwent MRI–TRUS fusion‐targeted transrectal prostate biopsy combined with systematic biopsy at the Fukui University Hospital between January 2022 and December 2024. The inclusion criteria were as follows: presence of suspected PCa lesions and a PI‐RADS score of 3 or higher on MRI examination. The exclusion criteria were as follows: prostate‐specific antigen (PSA) levels > 20 ng/mL, prior diagnosis of PCa, or suspected distant metastasis on imaging. Patients who underwent conventional systematic transrectal prostate biopsy at our institution between January 2019 and December 2020 served as the control group. First‐ and repeat‐biopsy patients were included in both cohorts. All clinical data were retrospectively extracted from electronic medical records.

### 2.2 Methods

MRI was performed using a 1.5T or 3.0T MRI scanner, and the images were evaluated based on PI‐RADS version 2.0. MRI–TRUS fusion‐targeted transrectal prostate biopsy was performed using the Trinity system (Koelis, Grenoble, France). Patients received prophylactic antibiotics prior to the biopsy, as either an intravenous injection of 1 g of cefmetazole or 4.5 g of piperacillin/tazobactam or as an oral administration of 500 mg of levofloxacin. All biopsies were performed transrectally using an 18‐gauge disposable core biopsy needle (Monopty MaxCore, BD, Franklin Lakes, NJ, USA) with a 22‐mm stroke length under caudal anesthesia. Targeted biopsies of two to three cores were performed for suspected PCa lesions. Systematic biopsies of 10 to 12 cores were also performed according to the operator's preference. The standard 10‐core scheme included bilateral base, middle, and apex regions, as well as bilateral lateral base and middle zones. In some cases, two additional cores were obtained from the bilateral TZ. The number of cores (10 or 12) was determined at the operator's discretion according to prostate size and the presence of TZ enlargement or ultrasound findings suggestive of nodular change. A schematic illustration of the systematic biopsy sites is provided as Figure S1. All biopsy procedures, including both conventional systematic and MRI–TRUS fusion‐targeted biopsies, were performed by 17 urologists whose clinical experience ranged from 3 to 15 years. In the control group, only systematic biopsy was performed. Among these patients, 25 had undergone prebiopsy MRI; however, they were not excluded from the main analysis. Cognitive targeting was permitted when suspicious lesions were recognized, although PI‐RADS scoring was not available in the radiology reports. In all of these 25 patients, MRI had been performed because PCa was clinically suspected based on elevated PSA levels, and systematic biopsy was subsequently conducted when MRI findings suggested malignancy. A sensitivity analysis excluding these 25 patients was also performed to confirm the robustness of the results.

Pathological diagnoses were performed by pathologists at Fukui University Hospital. The Gleason grade was determined using the International Society of Urological Pathology (ISUP) grading system. csPCa was defined as ISUP grade group 2 or higher, regardless of cancer core length. On the other hand, cisPCa was defined as anything other than csPCa.

### 2.3 Statistical Analysis

Continuous variables are presented as median and interquartile range (IQR), and differences between the groups were evaluated using the Student's *t*‐test. For categorical variables, differences were analyzed using the Fisher's exact test. Propensity score matching was performed using 1:1 nearest‐neighbor matching without replacement. A caliper width of 0.2 times the standard deviation of the propensity score (approximately 0.02 in this study) was used to balance baseline characteristics, including age, PSA levels, prostate volume, PSA density (PSAD), and first‐ or repeat‐biopsy status. Information on digital rectal examination and clinical T stage was unavailable for all patients and therefore not included in the propensity score model. Multivariate logistic regression analysis was also performed to identify independent predictors of clinically significant prostate cancer (csPCa) detection. The following variables were included in the model: PI‐RADS category, target lesion location (PZ, TZ/CZ, or both), PSAD, age, and biopsy status (initial or repeat). All analyses were performed using Easy R (EZR) (Saitama Medical Center, Jichi Medical University, Saitama, Japan), a graphical user interface for R (The R Foundation for Statistical Computing, Vienna, Austria) [11]. Statistical significance was defined as *p* < 0.05.

## 3 Results

### 3.1 Patient Characteristics

A total of 223 patients who underwent MRI–TRUS fusion‐targeted transrectal biopsy combined with systematic biopsy were included. The median age was 72 years (IQR, 66–75), the median PSA level was 8.0 ng/mL (IQR, 5.6–11.1), the median PSA density (PSAD) was 0.22 (IQR 0.15–0.35), and the median prostate volume was 35 mL (IQR, 25–51). An initial biopsy was performed in 161 patients (72%), and a repeat biopsy was performed in 62 patients (28%) (Table 1A).

**TABLE 1:** Patient characteristics in MRI–TRUS fusion and control groups.

(A) Before propensity score matching				
	MRI–TRUS fusion	Control	*p*	SMD
	*n* = 223	*n* = 161		
Age (years), median (IQR)	72 (66–75)	71 (66–77)	0.964	0.030
PSA (ng/mL), median (IQR)	8.0 (5.6–11.1)	7.2 (5.3–9.8)	0.053	0.189
Prostate volume (mL), median (IQR)	35 (25–51)	36 (28–53)	0.420	0.097
PSA density, median (IQR)	0.22 (0.15–0.35)	0.19 (0.14–0.28)	0.044	0.284
Initial biopsy, *n* (%)	161 (72)	137 (85)	0.003	0.319

(B) After propensity score matching				
	MRI–TRUS fusion	Control	*p*	SMD
	*n* = 147	*n* = 147		
Age (years), median (IQR)	73 (67–75)	72 (66–77)	0.553	0.111
PSA (ng/mL), median (IQR)	7.0 (5.4–9.9)	7.1 (5.3–9.5)	0.776	0.074
Prostate volume (mL), median (IQR)	35 (29–51)	34 (28–51)	0.400	0.071
PSA density, median (IQR)	0.19 (0.13–0.28)	0.20 (0.14–0.28)	0.568	0.012
Initial biopsy, *n* (%)	123 (84)	124 (84)	1.000	0.019

Abbreviations: IQR, interquartile range; MRI–TRUS, magnetic resonance imaging–transrectal ultrasound; PSA, prostate‐specific antigen; PSAD, PSA density; PSM, propensity score matching; SMD, standardized mean difference.

### 3.2 Cancer Detection Rates

The overall PCa detection rate was 67% (150/223). The csPCa and cisPCa detection rates were 60% (133/223) and 8% (17/223), respectively. The overall PCa, csPCa, and cisPCa detection rates were 75% (120/161), 63% (107/161), and 8% (13/161), respectively, in the initial biopsy group and 48% (30/62), 42% (26/62), and 6% (4/62), respectively, in the repeat‐biopsy group (Table 2).

**TABLE 2:** Prostate cancer detection rates (patient‐level).

	PCa rate	csPCa rate	cisPCa rate
	Positive/total (%)	Positive/total (%)	Positive/total (%)
Overall	150/223 (67)	133/223 (60)	17/223 (8)
Initial or repeat biopsy			
Initial biopsy	120/161 (75)	107/161 (63)	13/161 (8)
Repeat biopsy	30/62 (48)	26/62 (42)	4/62 (6)
Location of suspicious lesion			
PZ only	76/106 (72)	68/106 (64)	8/106 (7)
TZ/CZ only	57/96 (59)	50/96 (52)	7/96 (7)
PZ and TZ/CZ	17/21 (81)	15/21 (71)	2/21 (10)
Highest PI‐RADS category, *n* (%)			
3	37/87 (43)	29/87 (33)	8/87 (9)
4	94/113 (83)	85/113 (75)	9/113 (8)
5	19/23 (83)	19/23 (83)	0/23 (0)

Abbreviations: cisPCa, clinically insignificant prostate cancer; csPCa, clinically significant prostate cancer; PCa, prostate cancer; PI‐RADS, Prostate Imaging Reporting and Data System; PZ, peripheral zone; TZ/CZ, transitional/central zones.

According to the location of the MRI‐identified target lesions, PCa was detected in 76/106 (72%) patients with lesions located only in the PZ, 57/96 (59%) with lesions only in the TZ/CZ, and 17/21 (81%) with lesions in both the PZ and TZ/CZ. In the PZ, TZ/CZ, and PZ plus TZ/CZ regions, the csPCa detection rates were 68/106 (64%), 50/96 (52%), and 15/21 (71%), respectively. In the same manner, the respective cisPCa detection rates were 8/106 (7%), 7/96 (7%), and 2/21 (10%) (Table 2).

When stratified by the highest PI‐RADS score, PCa was detected in 37/87 (43%) patients with PI‐RADS 3 lesions, 94/113 (83%) with PI‐RADS 4 lesions, and 19/23 (83%) with PI‐RADS 5 lesions. For PI‐RADS scores of 3, 4, and 5, the respective csPCa detection rates were 29/87 (33%), 85/113 (75%), and 19/23 (83%), whereas the respective cisPCa detection rates were 8/87 (9%), 9/113 (8%), and 0/23 (0%) (Table 2).

### 3.3 Lesion‐Level Cancer Detection by PI‐RADS Category and Lesion Location

At the lesion level, cancer detection rates according to PI‐RADS category and target lesion location are summarized in Table 3. Detection rates for both overall and csPCa increased with higher PI‐RADS categories. The detection rates were generally lower for lesions located in the TZ/CZ than for those in the PZ. For PI‐RADS 3, 4, and 5 lesions, the detection rates of any cancer in the PZ were 15/46 (33%), 64/90 (71%), and 8/8 (100%), respectively, and those in the TZ/CZ were 20/87 (23%), 25/41 (61%), and 11/15 (73%), respectively. For csPCa, the corresponding rates in the PZ were 10/46 (22%), 59/90 (66%), and 8/8 (100%), and those in the TZ/CZ were 14/87 (16%), 22/41 (54%), and 10/15 (67%), respectively.

**TABLE 3:** Prostate cancer detection rates (lesion‐level).

(A) Any prostate cancer (lesion‐level)			
PI‐RADS category	PZ	TZ/CZ	All lesions
	Positive/total (%)	Positive/total (%)	Positive/total (%)
3	15/46 (33)	20/87 (23)	35/133 (25)
4	64/90 (71)	25/41 (61)	89/131 (68)
5	8/8 (100)	11/15 (73)	19/23 (83)

(B) Clinically significant cancer (ISUP ≥ 2, lesion‐level)			
PI‐RADS category	PZ	TZ/CZ	All lesions
	Positive/total (%)	Positive/total (%)	Positive/total (%)
3	10/46 (22)	14/87 (16)	24/133 (18)
4	59/90 (66)	22/41 (54)	81/131 (62)
5	8/8 (100)	10/15 (67)	18/23 (78)

Abbreviations: ISUP, International Society of Urological Pathology; PI‐RADS, Prostate Imaging Reporting and Data System; PZ, peripheral zone; TZ/CZ, transitional/central zones.

### 3.4 Multivariate Analysis for Predictors of csPCa Detection

Multivariate logistic regression analysis was performed to identify independent predictors of csPCa detection on a per‐patient basis (Table 4). Higher PI‐RADS category and PSA density were independent predictors of csPCa detection (both *p* < 0.001). Lesions located exclusively in the TZ/CZ were less likely to yield csPCa than those in the PZ (odds ratio 0.48, 95% CI 0.23–0.98, *p* = 0.044). Age and biopsy status (initial vs. repeat) were not significantly associated with csPCa detection.

**TABLE 4:** Multivariable logistic regression analysis for predictors of csPCa detection (patient‐level).

Variable	Adjusted OR	95% CI	*p*
PI‐RADS 4 vs. 3	4.26	2.07–8.78	< 0.001
PI‐RADS 5 vs. 3	15.1	4.34–52.5	< 0.001
Only TZ/CZ vs. only PZ	0.48	0.23–0.98	0.044
Both vs. only PZ	0.53	0.17–1.67	0.278
PSA density (per 0.1)	1.83	1.44–2.32	< 0.001
Age (per year)	1.01	0.96–1.06	0.763
Initial vs. repeat biopsy	1.91	0.91–4.02	0.089

Abbreviations: csPCa, clinically significant prostate cancer; PCa, prostate cancer; PI‐RADS, Prostate Imaging Reporting and Data System; PZ, peripheral zone; TZ/CZ, transitional/central zones.

### 3.5 Efficacy and Safety Compared With Conventional Systematic Biopsy

To evaluate the efficacy and safety of MRI–TRUS fusion‐targeted biopsy in addition to systematic biopsy (MRI–TRUS fusion group), 161 patients who had previously undergone only systematic biopsy were included as a control group. Among these 161 patients, 25 had undergone prebiopsy MRI; however, they were not excluded from the main analysis. The results remained consistent in a sensitivity analysis excluding these cases, indicating that inclusion of these patients did not affect the overall findings (Table S1). Age, PSA levels, and prostate volume were not significantly different between the groups. PSA density was significantly higher (*p* = 0.044), and the proportion of patients undergoing initial biopsy was significantly lower in the MRI–TRUS fusion group than in the control group (72% vs. 85%, *p* = 0.003) (Table 1A).

The overall PCa detection rate (67% vs. 46%, *p* < 0.001) and csPCa detection rates (60% vs. 39%, *p* < 0.001) were significantly higher in the MRI–TRUS fusion group than in the control group. However, the cisPCa detection rates were not significantly different (8% vs. 8%, *p* = 1.000) between the two groups (Table 5A).

**TABLE 5:** Cancer detection rates in MRI–TRUS fusion and control groups.

(A) Before propensity score matching				
Outcome	MRI–TRUS fusion	Control	*p*	SMD
	*n* = 223	*n* = 161		
Overall PCa detection, *n* (%)	150 (67)	75 (46)	< 0.001	0.427
csPCa detection, *n* (%)	133 (60)	62 (39)	< 0.001	0.432
cisPCa detection, *n* (%)	17 (8)	13 (8)	1.000	0.017

(B) After propensity score matching				
Outcome	MRI–TRUS fusion	Control	*p*	SMD
	*n* = 147	*n* = 147		
Overall PCa detection, *n* (%)	97 (66)	72 (49)	0.005	0.349
csPCa detection, *n* (%)	85 (58)	60 (41)	0.005	0.345
cisPCa detection, *n* (%)	12 (8)	12 (8)	1.000	< 0.001

Abbreviations: cisPCa, clinically insignificant prostate cancer; csPCa, clinically significant prostate cancer; MRI–TRUS, magnetic resonance imaging–transrectal ultrasound; PCa, prostate cancer; PSM, propensity score matching; SMD, standardized mean difference.

Propensity score matching was used to adjust for differences in baseline characteristics between the groups, as this approach allows for a more balanced comparison of diagnostic outcomes. After propensity score matching, 147 patients were included in each cohort with balanced baseline characteristics, including age (73 vs. 72 years, *p* = 0.553), PSA levels (7.0 vs. 7.1 ng/mL, *p* = 0.776), prostate volume (35 vs. 34 mL, *p* = 0.400), PSA density (0.19 vs. 0.20, *p* = 0.568) and initial biopsy rates (84% vs. 84%, *p* = 1.000) (Table 1B). In the matched cohorts, the overall PCa detection rate (66% vs. 49%, *p* = 0.005) and csPCa detection rates (58% vs. 41%, *p* = 0.005) were significantly higher in the MRI–TRUS fusion group than in the control group. The cisPCa detection rates were not significantly different between the groups (8% vs. 8%, *p* = 1.000) (Table 5B).

The occurrence rate of adverse events including infection, rectal bleeding, and urinary retention was compared between the groups. The rate of grade 3 or higher adverse events was not significantly different between MRI–TRUS fusion group and the control group (2% vs. 3%, *p* = 0.500) (Table 6A). A similar trend was observed after propensity score matching (3% vs. 3%, *p* = 1.000) (Table 6B).

**TABLE 6:** Adverse events (grade ≥ 3) in MRI–TRUS fusion and control groups.

(A) Before propensity score matching				
	MRI–TRUS fusion	Control	*p*	SMD
	*n* = 223	*n* = 161		
≥ Grade 3 adverse events, *n* (%)	4 (1.8)	5 (3.1)	0.500	0.085
Infection requiring hospitalization (acute prostatitis, febrile UTI), *n* (%)	2 (0.9)	1 (0.6)	1.000	0.032
Rectal bleeding requiring endoscopic or radiologic intervention, *n* (%)	1 (0.4)	2 (1.2)	0.574	0.087
Urinary retention requiring hospitalization, *n* (%)	1 (0.4)	2 (1.2)	0.574	0.087

(B) After propensity score matching				
	MRI–TRUS fusion	Control	*p*	SMD
	*n* = 147	*n* = 147		
≥ Grade 3 adverse events, *n* (%)	4 (2.7)	4 (2.7)	1.000	< 0.001
Infection requiring hospitalization (acute prostatitis, febrile UTI), *n* (%)	2 (1.4)	1 (0.7)	1.000	0.068
Rectal bleeding requiring endoscopic or radiologic intervention, *n* (%)	1 (0.7)	2 (1.4)	1.000	0.068
Urinary retention requiring hospitalization, *n* (%)	1 (0.7)	1 (0.7)	1.000	< 0.001

Abbreviations: MRI–TRUS, magnetic resonance imaging–transrectal ultrasound; PSM, propensity score matching; SMD, standardized mean difference; UTI, urinary tract infection.

## 4 Discussion

In this study, compared with conventional systematic biopsy, MRI–TRUS fusion‐targeted transrectal biopsy combined with systematic biopsy resulted in significantly higher overall PCa and csPCa detection rates, without increasing the rates of cisPCa detection or adverse events. These findings are consistent with those of previous reports showing the superior diagnostic performance of MRI‐targeted biopsy, reinforcing its clinical value [8, 9, 10].

Importantly, because this was a retrospective study, we applied propensity score matching to adjust for baseline differences between the MRI–TRUS fusion and control groups, including age, PSA levels, prostate volume, PSA density, and initial or repeat biopsies. Even after matching, the MRI–TRUS fusion group retained a significant advantage in overall PCa and csPCa detection. Most previous studies included only patients who underwent an initial biopsy for biopsy‐naïve populations and did not apply propensity score adjustment. Our study of both initial‐ and repeat‐biopsy patients provides a more representative reflection of the real‐world clinical practice.

We evaluated the PCa and csPCa detection rates for each PI‐RADS score. In the PRECISION study, PCa and csPCa detection rates were 34% and 12% in PI‐RADS 3, 69% and 60% in PI‐RADS 4, and 94% and 83% in PI‐RADS 5, respectively [6]. Fujii et al. reported PCa and csPCa detection rates of 38% and 19% for PI‐RADS 3, 85% and 67% for PI‐RADS 4, and 91% and 70% for PI‐RADS 5, respectively [9]. In our study, the PCa and csPCa detection rates were 43% and 33% in PI‐RADS 3, 83% and 75% in PI‐RADS 4, and 83% and 83% in PI‐RADS 5, respectively, consistent with previous reports (Table S2). Based on these results, the indications for prostate biopsy in patients with PI‐RADS 3 lesions should be reconsidered, given the moderate csPCa detection rate in such lesions. Biopsy decisions should be individualized, considering PSA density and overall clinical context.

We found that the detection rate of PCa tended to be lower for lesions only in the TZ/CZ (59%) than for lesions only in the PZ (72%) or both the PZ and TZ/CZ (81%). These findings are consistent with those of previous studies that reported lower detection rates in the TZ/CZ (52%) than in the PZ (76%) [9, 10]. One possible explanation is the heterogeneous tissue composition of the TZ/CZ, which can complicate the differentiation of PCa from benign conditions on MRI [5, 12]. Additionally, the longer puncture distance required to reach the TZ/CZ lesions compared to the PZ lesions may have increased the risk of deviation from the target. Improvements in imaging technology, PI‐RADS interpretation, and operator training are essential to enhance the diagnostic accuracy in these challenging regions.

MRI–TRUS fusion‐targeted biopsies were performed using the transrectal approach. In a study comparing the efficacy of transperineal and transrectal MRI‐targeted prostate biopsies, PCa and csPCa detection rates were higher for transperineal than for transrectal biopsies [13]. As a multi‐institutional retrospective study, the biopsy methods differed among institutes, resulting in lower detection rates than those in our study. In a meta‐analysis, the PCa and csPCa detection rates did not differ significantly between transperineal and transrectal MRI‐targeted prostate biopsies [14]. An MRI–TRUS fusion‐targeted transrectal biopsy can be performed under caudal anesthesia. Adverse events, such as acute prostatitis or rectal bleeding, occurred in only 3% of cases, which was not higher than the rate observed with conventional systematic biopsy. These results indicate that MRI–TRUS fusion‐targeted transrectal biopsy is effective for csPCa diagnosis and can be safely performed.

Our diagnostic procedures included MRI–TRUS fusion‐targeted and systematic biopsies. Blas et al. reported that MRI–TRUS fusion‐targeted biopsy alone missed approximately 5% of csPCa cases [9]. Similarly, Ahdoot et al. demonstrated that combining MRI–TRUS fusion‐targeted biopsy with systematic biopsy significantly improved cancer detection compared with either approach alone [15]. In our study, 24 of the 150 PCa (16%) and 19 of the 133 csPCa (14%) cases were detected only by systematic biopsy, and 21 PCa (14%) and 16 csPCa (12%) cases were detected only by MRI–TRUS fusion‐targeted biopsy. These findings indicate that MRI–TRUS fusion‐targeted biopsy alone is insufficient for an accurate diagnosis and suggest that combined biopsy methods may improve the diagnostic yield. Future enhancements in MRI resolution, lesion interpretation, and PI‐RADS standardization may improve the stand‐alone utility of MRI–TRUS fusion‐targeted biopsies.

This study had several limitations. Inherent to a retrospective design and single‐institution analysis, selection bias might limit the generalizability of the findings. Although propensity score matching was used to balance key baseline characteristics, we were unable to include MRI‐derived variables, such as the PI‐RADS score, owing to the lack of MRI data in the systematic biopsy group. Some patients in the control group had undergone prebiopsy MRI, and cognitive targeting was permitted in those cases. However, the number of such patients was limited (*n* = 25), and PI‐RADS‐based interpretation was not applied. When these patients were excluded from the analysis, the results remained consistent with the main findings, suggesting that the inclusion of these cases did not materially affect the study conclusions. Consequently, residual confounding factors may have influenced the outcomes. In addition, MRI interpretation and biopsy performance are operator‐dependent, introducing potential variability in diagnostic accuracy. Furthermore, inter‐reader variability in the PI‐RADS assessment was not evaluated and may have affected the lesion classification [16]. Finally, the study period was extended over several years, during which advances in imaging technology and operator expertise may have impacted diagnostic performance. Future prospective multicenter studies with standardized imaging protocols and centralized radiological reviews are essential to confirm the findings in broader clinical settings.

In conclusion, MRI–TRUS fusion‐targeted transrectal biopsy combined with systematic biopsy significantly improved overall and csPCa detection compared with systematic biopsy alone. By including both initial‐ and repeat‐biopsy patients, this study extends the previous knowledge base and underscores the broad applicability of MRI–TRUS fusion‐targeted biopsy.

## Author Contributions

**Yusuke Fukiage:** conceptualization, methodology, data curation, formal analysis, investigation, writing – original draft. **Minekatsu Taga:** writing – review and editing, supervision. **Mahiro Inamura:** investigation. **Kimika Kaeriyama:** investigation. **Nobuki Tanaka:** investigation. **Wonseok Seo:** investigation. **Tadashi Kakitsuba:** investigation. **Sahoko Shimada:** investigation. **Nodoka Okubo:** investigation. **Takafumi Kabuto:** investigation. **Manami Tsutsumiuchi:** investigation. **Yoshinaga Okumura:** investigation. **Akifumi Muramoto:** investigation. **Masaya Seki:** investigation. **So Inamura:** investigation. **Naoki Terada:** supervision.

## Funding

The authors have nothing to report.

## Ethics Statement

This study was approved by the Ethics Committee of the Faculty of Medical Sciences, University of Fukui (reference number: 20190174; approved on March 18, 2020). This retrospective study was conducted under this approval, with an additional amendment obtained before data collection.

## Consent

Instead of obtaining individual written informed consent from patients, an opportunity to opt out was provided.

## Conflicts of Interest

Naoki Terada is an Editorial Board member of *International Journal of Urology* and a co‐author of this article. To minimize bias, he was excluded from all editorial decision‐making related to the acceptance of this article for publication.

## Supporting information


**Figure S1:** Schematic illustration of the systematic prostate biopsy sites. Systematic biopsy cores were obtained from the bilateral base, middle, and apex regions, as well as the bilateral lateral base and middle zones. In some cases, two additional cores were taken from the bilateral transitional zones (TZ).

**Table S1:** Patient characteristics and cancer detection rates in MRI–TRUS fusion and control groups.
**Table S2:** Detection rates in PI‐RADS 3 lesions (comparison with previous studies).

## Data Availability

The datasets generated and analyzed during the current study are not publicly available but are available from the corresponding author on reasonable request.
