# Supplementary figures and images for: Magnetic Resonance Imaging–Transrectal Ultrasound Fusion‐Targeted Biopsy Improves the Diagnostic Efficacy of Overall and Clinically Significant Prostate Cancer
Source: Int J Urol. 2025 Dec 26;33(1):e70334. doi: 10.1111/iju.70334 (PMC12828291; doi:10.1111/iju.70334)

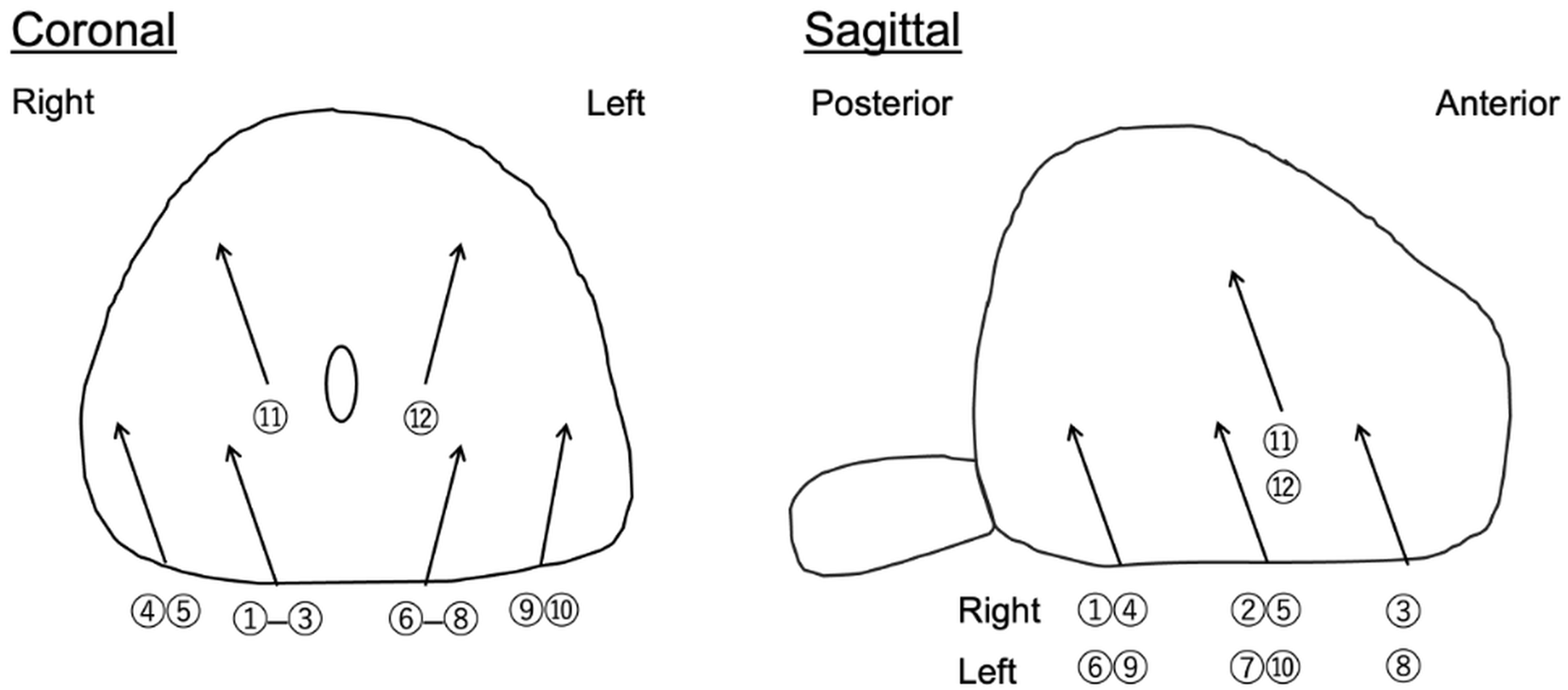

Supplement: Supplementary file 1 — Figure S1: Schematic illustration of the systematic prostate biopsy sites. Systematic biopsy cores were obtained from the bilateral base, middle, and apex regions, as well as the bilateral lateral base and middle zones. In some cases, two additional cores were taken from the bilateral transitional zones (TZ). [file IJU-33-0-s001.tiff]
