# Supplementary material for: Magnetic Resonance Imaging–Transrectal Ultrasound Fusion‐Targeted Biopsy Improves the Diagnostic Efficacy of Overall and Clinically Significant Prostate Cancer
Source: Int J Urol. 2025 Dec 26;33(1):e70334. doi: 10.1111/iju.70334 (PMC12828291; doi:10.1111/iju.70334)
Supplement: Supplementary file 2 — Table S1: Patient characteristics and cancer detection rates in MRI–TRUS fusion and control groups. Table S2: Detection rates in PI‐RADS 3 lesions (comparison with previous studies). [file IJU-33-0-s002.docx]

**Table S1. Patient characteristics and cancer detection rates in MRI-TRUS fusion and control groups**

(A) Before propensity score matching

|  | MRI-TRUS fusion | Control | *p*-value | SMD |
| --- | --- | --- | --- | --- |
|  | *n* = 223 | *n* = 136 |  |  |
| Age (years), median (IQR) | 72 (66–75) | 71 (65–77) | 0.662 | 0.072 |
| PSA (ng/mL), median (IQR) | 8.0 (5.7–11.1) | 7.1 (5.3–9.8) | 0.027 | 0.236 |
| Prostate volume (mL), median (IQR) | 35 (25–51) | 36 (28–53) | 0.425 | 0.104 |
| PSA density, median (IQR) | 0.22 (0.15–0.35) | 0.19 (0.14–0.26) | 0.029 | 0.323 |
| Initial biopsy, *n* (%) | 161 (72) | 119 (88) | 0.001 | 0.389 |
| Overall PCa detection, *n* (%) | 150 (67) | 59 (43) | <0.001 | 0.495 |
| csPCa detection, *n* (%) | 133 (60) | 47 (35) | <0.001 | 0.519 |
| cisPCa detection, *n* (%) | 17 (8) | 12 (9) | 0.694 | 0.044 |

(B) After propensity score matching

|  | MRI-TRUS fusion | Control | *p*-value | SMD |
| --- | --- | --- | --- | --- |
|  | *n* = 126 | *n* = 126 |  |  |
| Age (years), median (IQR) | 72 (67–75) | 72 (66–77) | 0.926 | 0.030 |
| PSA (ng/mL), median (IQR) | 7.0 (5.4–9.9) | 7.1 (5.3–10.0) | 0.981 | 0.030 |
| Prostate volume (mL), median (IQR) | 36 (29–55) | 34 (27–52) | 0.158 | 0.169 |
| PSA density, median (IQR) | 0.18 (0.13–0.26) | 0.20 (0.15–0.27) | 0.175 | 0.090 |
| Initial biopsy, n (%) | 106 (84) | 109 (87) | 0.722 | 0.067 |
| Overall PCa detection, *n* (%) | 81 (64) | 58 (46) | 0.005 | 0.373 |
| csPCa detection, *n* (%) | 72 (57) | 47 (37) | 0.002 | 0.406 |
| cisPCa detection, *n* (%) | 9 (7) | 11 (9) | 0.816 | 0.059 |

| *Abbreviations:* cisPCa, clinically insignificant prostate cancer; csPCa, clinically significant prostate cancer; PCa, prostate cancer; MRI-TRUS, magnetic resonance imaging-transrectal ultrasound; PSA, prostate-specific antigen; PSAD, PSA density; IQR, interquartile range; PSM, propensity score matching; SMD, standardized mean difference. |  |
| --- | --- |
|  |  |

**Table S2. Detection rates in PI-RADS 3 lesions (comparison with previous studies)**

| Study | Biopsy method | PCa detection | csPCa detection | | |
| --- | --- | --- | --- | --- | --- |
| PRECISION | Targeted biopsy only | 34% | 12% | | |
| Fujii et al. | Systematic + targeted biopsy | 38% | 19% | | |
| Current study | Systematic + targeted biopsy | 43% | 33% | | |
| *Abbreviations*: csPCa, clinically significant prostate cancer; PCa, prostate cancer; PI-RADS, Prostate Imaging Reporting and Data System. | | | |  |  |
|  |  |  |  |  |  |
